# Repurposing Cationic Amphiphilic Antihistamines for Cancer Treatment

**DOI:** 10.1016/j.ebiom.2016.06.013

**Published:** 2016-06-07

**Authors:** Anne-Marie Ellegaard, Christian Dehlendorff, Anna C. Vind, Atul Anand, Luise Cederkvist, Nikolaj H.T. Petersen, Jesper Nylandsted, Jan Stenvang, Anders Mellemgaard, Kell Østerlind, Søren Friis, Marja Jäättelä

**Affiliations:** a Cell Death & Metabolism, Center for Autophagy, Recycling and Disease, Danish Cancer Society Research Center, DK-2100 Copenhagen, Denmark; b Statistics, Bioinformatics & Registry, Danish Cancer Society Research Center, DK-2100 Copenhagen, Denmark; c Department of Veterinary Disease Biology, Section for Molecular Disease Biology, Faculty of Health and Medical Sciences, Copenhagen University, DK-2100 Copenhagen, Denmark; d Department of Oncology, Copenhagen University Hospital Herlev, DK-2730 Herlev, Denmark; e Department of Oncology, Copenhagen University Hospital Rigshospitalet, DK-2200 DK-2730 Copenhagen, Denmark

## Abstract

Non-small cell lung cancer (NSCLC) is one of the deadliest cancers worldwide. In search for new NSCLC treatment options, we screened a cationic amphiphilic drug (CAD) library for cytotoxicity against NSCLC cells and identified several CAD antihistamines as inducers of lysosomal cell death. We then performed a cohort study on the effect of CAD antihistamine use on mortality of patients diagnosed with non-localized cancer in Denmark between 1995 and 2011. The use of the most commonly prescribed CAD antihistamine, loratadine, was associated with significantly reduced all-cause mortality among patients with non-localized NSCLC or any non-localized cancer when compared with use of non-CAD antihistamines and adjusted for potential confounders. Of the less frequently described CAD antihistamines, astemizole showed a similar significant association with reduced mortality as loratadine among patients with any non-localized cancer, and ebastine use showed a similar tendency. The association between CAD antihistamine use and reduced mortality was stronger among patients with records of concurrent chemotherapy than among those without such records. In line with this, sub-micromolar concentrations of loratadine, astemizole and ebastine sensitized NSCLC cells to chemotherapy and reverted multidrug resistance in NSCLC, breast and prostate cancer cells. Thus, CAD antihistamines may improve the efficacy of cancer chemotherapy.

## Highlights

• Use of cationic amphiphilic antihistamines is associated with reduced mortality among patients with non-localized cancer.
• Clinically relevant concentrations of cationic amphiphilic antihistamines sensitize cancer cells to chemotherapy.
• Clinically relevant concentrations of cationic amphiphilic antihistamines revert multidrug resistance.
  **Research Context** Cationic amphiphilic drugs (CADs) induce lysosomal membrane permeabilization and cell death preferentially in cancer cells. Here, we show that antihistamines with CAD structure, *i.e.* astemizole, ebastine and loratadine, sensitize cancer cells to chemotherapy and revert multidrug resistance even at low, clinically relevant concentrations. The significance of these experimental findings is supported by an association between CAD antihistamine use and reduced mortality among patients diagnosed with non-localized cancer, especially among those receiving concurrent chemotherapy. These findings are immediately translatable to clinical trials, as loratadine and ebastine, are safe, inexpensive and approved for clinical use.

## 1 Introduction

Non-small cell lung cancer (NSCLC) is one of the most common cancers and the leading cause of cancer death worldwide (Siegel et al., 2015). The majority of patients are diagnosed only after the disease has spread beyond the primary site. Thus, systemic chemotherapy, usually with combinations containing platinum-based and microtubule-disturbing drugs, forms the foundation of the treatment of these patients. As is the case for most advanced cancers, acquired apoptosis and therapy resistance pose, however, major challenges for the treatment of NSCLC (Chang, 2011). During cancer development, cells accumulate numerous genetic and epigenetic alterations to escape apoptosis initially induced by the transformation process itself, later by the hostile tumor environment and finally by cancer treatment (Groth-Pedersen and Jäättelä, 2013, Hanahan and Weinberg, 2011). Moreover, chemotherapy-treated cancer cells often acquire an ability to efflux the chemotherapeutic drugs by increasing the expression of multidrug resistance (MDR)-associated P-glycoproteins of the ATP-binding cassette transporter family (Gottesman et al., 2002, Chang, 2011). Importantly, cells harbor alternative cell death pathways that remain functional even in otherwise therapy-resistant cancer cells (Fulda, 2014, Kallunki et al., 2013). Of special interest in this context is lysosomal cell death. Cancer progression to metastatic disease depends on the activation of the lysosomal compartment, which is manifested by increased lysosomal biogenesis and acidification (Kallunki et al., 2013, Perera et al., 2015). Besides being tumor-promoting, these lysosomal changes associate with reduced lysosomal membrane stability (Fehrenbacher et al., 2008, Fehrenbacher et al., 2004). This frailty of cancer cell lysosomes can be targeted by several cationic amphiphilic drugs (CADs) that accumulate in the acidic lysosomes and induce lysosomal damage preferentially in cancer cells (Ostenfeld et al., 2008, Petersen et al., 2013, Sukhai et al., 2013, Jahchan et al., 2013, Shchors et al., 2015).

CADs include hundreds of pharmacologic agents used to treat a broad spectrum of common diseases, *e.g.* psychiatric disorders, allergies, heart diseases and infections (Kornhuber et al., 2010). They are characterized by a hydrophobic ring structure and a hydrophilic side chain with a cationic amine group. In acidic milieu, the basic amine groups are protonated allowing an up to 1000-fold drug accumulation inside acidic lysosomes (Trapp et al., 2008). The incorporation of CADs into membranes in the lysosomal lumen neutralizes the negative membrane charge thereby inhibiting the function of several lysosomal lipases, including acid sphingomyelinase (Kolzer et al., 2004). Cancer cells are especially sensitive to the accumulation of sphingomyelin (Barcelo-Coblijn et al., 2011, Teres et al., 2012, Petersen et al., 2013), which may explain why CADs that are effective acid sphingomyelinase inhibitors display selective cytotoxicity towards transformed cells (Petersen et al., 2013, Sukhai et al., 2013, Jahchan et al., 2013, Shchors et al., 2015).

Repurposing of well-characterized and well-tolerated drugs for cancer therapy has emerged as an attractive alternative for a long and costly process of drug development. Encouraged by the well-documented anti-cancer activity of several CADs, we searched systematically for CADs with highest anti-NSCLC potential by screening a CAD library for cytotoxicity against A549 NSCLC cells. Prompted by the enrichment of antihistamines among the hits, we performed a more detailed study of their cytotoxic activity alone and in combination with chemotherapy, and conducted a pharmacoepidemiological register-based cohort study of the association between CAD antihistamine use and mortality among Danish cancer patients.

## 2 Materials and Methods

### 2.1 Pharmacoepidemiological Study

To evaluate the association between use of antihistamines and mortality among all Danish residents above 30 years of age diagnosed with any non-localized cancer (defined based on either regional or distant metastases) during 1995–2011 or non-localized NSCLC during 2004–2011 (Supplemental Table S1), we linked data from six nationwide sociodemographic or health registries described below and in the Supplemental Table S2 using the personal identification number assigned to all Danish residents (Thygesen et al., 2011). From the Danish Prescription Registry, we retrieved information on prescriptions dispensed during 1995–2011 for systemic CAD (astemizole, clemastine, desloratadine, ebastine, loratadine and terfenadine) and non-CAD (cetirizine and fexofenadine) antihistamines (Supplemental Table S2). Ebastine, loratadine, cetirizine and fexofenadine became available over-the-counter during the study period. The majority of the antihistamine sale (ebastine > 75%, loratadine > 65%, cetirizine > 55% and fexofenadine > 97%) was, however, by prescription (Sundhedsdatastyrelsen, 2016). We defined antihistamine (CAD or non-CAD) use as one or more prescriptions within 0–6 month following the diagnosis of any non-localized cancer and from three months before until three months after the non-localized NSCLC diagnosis. The patients were followed from six (all non-localized cancers) or three (non-localized NSCLC) months after the diagnosis until death, emigration, or end of study (31 December 2013), whichever occurred first. Cox proportional hazards regression was used to estimate hazard ratios (HRs) and 95% confidence intervals (CIs) for all-cause mortality associated with the use of antihistamines. The time since baseline was used as the underlying time-scale. We compared users of CAD antihistamines with non-users, as well as with users of either of the two non-CAD antihistamines fexofenadine or cetirizine, while adjusting for covariates identified from prescription and patient registries (Tables S3 and S4). We repeated the analyses stratified according to records of chemotherapy (yes/no) during the first six months following the diagnosis, which were available only for patients diagnosed between 2002–2011.

The HR estimates for all-cause death associated with use of antihistamine were adjusted for age, year of cancer diagnosis, highest achieved education, disposable income, Charlson Comorbidity Index score and drugs as described below. From the Prescription Registry (Kildemoes et al., 2011), we obtained information on prescriptions of aspirin, non-aspirin nonsteroidal anti-inflammatory drugs (NA-NSAID), statins and inhibitors of the renin-angiotensin system (including angiotensin converting enzyme inhibitors (ACEi) and angiotensin-receptor blockers (ARB)). Use of the ‘confounder drugs’ was defined as ≥ 1 prescriptions within the exposure period for antihistamines. From the Danish National Patient Registry (Schmidt et al., 2015), we retrieved information on history (at baseline) of diagnoses of chronic conditions included in the validated Charlson Comorbidity Index (Charlson et al., 1987) and computed Charlson Comorbidity Index score, categorized as 0, 1 or ≥ 2. Socio-economic status one year prior to the cancer diagnosis was estimated by the highest achieved education and the disposable income retrieved from registers at Statistics Denmark (Jensen and Rasmussen, 2011, Baadsgaard and Quitzau, 2011).

The local institutional review board and the Danish Data Protection Agency approved the study and waived the requirement for individual informed consent. Ethical approval is not required for registry-based studies in Denmark.

### 2.2 Danish Registries

The *Danish Cancer Registry* has recorded detailed nationwide information on cancer incidence since 1943 and offers an accurate and almost complete record of cancer cases (Storm et al., 1997, Gjerstorff, 2011). Cancer diagnoses are recorded according to the *International Classification of Diseases*, Eighth (ICD-8) or Tenth Revision (ICD-10), and the *International Classification of Diseases for Oncology* (ICD-O) is used for coding of topography and morphology (Gjerstorff, 2011). The Cancer Registry also contains data on clinical stage, categorized as localized, regional, distant, or unknown until 2003 and according to the tumor-node-metastasis (TNM) system from 2004 to the present (Storm et al., 1997, Gjerstorff, 2011, Edge and COMPTON, 2010).

The *Danish Prescription Registry* consists of records of all drug prescriptions dispensed at pharmacies in Denmark since 1995 (Kildemoes et al., 2011). The data include the type and amount of drug prescribed according to the Anatomical Therapeutical Chemical (ATC) classification system (WHO, 2013), number of packages, and the date of dispensing at the pharmacy. The dosing schedule and indication(s) are not recorded, and no information is available on drug use dispensed at hospital level.

*The Danish National Patient Registry* contains detailed individual data on all somatic hospitalizations in Denmark since 1977 and on ambulatory hospital contacts and psychiatric admissions since 1995 (Schmidt et al., 2015). Discharge and contact diagnoses are coded according to ICD-8 from 1977 to 1993 and ICD-10 from 1994 to the present. Information on main types of oncological therapy (chemotherapy, radiotherapy, endocrine therapy, *etc.*) is available from 2002.

The *Danish Register of Causes of Death* contains information on date and cause of death of all inhabitants of Denmark, classified according to ICD-8 until 1993 and to ICD-10 from 2004 (Helweg-Larsen, 2011).

*Statistics Denmark* administers registries on socio-economic data, including education and income, of all Danish residents (Jensen and Rasmussen, 2011, Baadsgaard and Quitzau, 2011).

The *Population Education Register* holds information on the highest completed level of education, derived from type and duration of schooling (Baadsgaard and Quitzau, 2011).

The *Danish Civil Registration System* maintains the civil registry number (encoding gender and date of birth) assigned to all Danish residents since 1968 and contains continuously updated address, date of death, and migration to and from Denmark. Use of the civil registration number ensures unambiguous linkage between population-based registries (Thygesen et al., 2011, Schmidt et al., 2014).

### 2.3 Cell Culture and Treatments

A549 (ATCC® CCL-185™), NCI-H1299 (ATCC® CRL-5803™) and NCI-H661 (ATCC® HTB-183™) NSCLC cell lines, DU145 prostate cancer cell line (ATCC® HTB-81™) and MDA-MB-231 breast cancer cell line (ATCC® HTB-26™) were obtained from American Type Culture Collection (ATCC). The cells were authenticated by the ATCC by short tandem repeat analysis, and they were used within 6 months after thawing. Multidrug-resistant variants of DU145 cells (DU145-MDR) and MDA-MB-231 (MDA-MB-231-MDR) have been described previously (Ellegaard et al., 2013, Hansen et al., 2015). The multidrug resistant variant of A549 cells (A549-MDR) were derived by repeated 3-day treatments of A549 cells with increasing doses of vinorelbine up to 150 nM. The parental cells (DU145-P, MDA-MB-231-P and A549-P, respectively) were grown in parallel. The NIH-3T3 fibroblasts transduced with either the empty pBabe-puro retrovirus or the c-Src^Y527F^-containing pBabe-puro retrovirus have been described elsewhere (Fehrenbacher et al., 2004), and their identity has been confirmed with RNA-Seq (Petersen et al., 2013). The A549, NCI-H661, NCI-H1299 and DU145 cells were cultured in RPMI-1640 (Gibco, 61870-010) supplemented with 10% (A549, NCI-H661 and NCI-H1299) or 6% (DU145) heat-inactivated fetal calf serum (Gibco, 10270). The MDA-MB-231 and NIH-3T3 cells were cultured in Dulbecco's Modified Eagle's medium (Gibco, 31966-021) supplemented with 10% heat-inactivated fetal calf serum and for the NIH-3T3 cells also with non-essential amino acids (Gibco, 11140-035). All cells were kept at 37 °C in a humidified atmosphere of 5% CO_2_. All cells were regularly tested and found negative for mycoplasma.

The providers, catalog numbers, and CAS numbers of the compounds in the CAD library are listed in Supplemental Table S5. *O*-desmethyl-astemizole (D290750) was purchased from Toronto Research Chemicals, cisplatin (P4394), cetirizine (C3618), propidium iodide (P4864), necrostatin-1 (N9037), docetaxel (01885), vinorelbine ditartrate salt (V2264), fexofenadine hydrochloride (F9427), ebastine (E9531), KO143 (K2144) and Hoechst-33342 (B2261) from Sigma; benzyloxycarbonyl-Val-Ala-Asp (OMe)fluoromethylketone (zVAD-fmk) (N1510-0025) from Bachem; leucin-leucin-*O*-methyl (LLOMe) (sc-285992) and carebastine (sc-211022) from Santa Cruz Biotechnology; PSC833 (ab145870) from Abcam; cyclizine hydrochloride (C3090000) from European Pharmacopoeia Reference Standard; and meclizine dihydrochloride (155341) from MP Biochemicals. Siramesine was kindly provided by Christiane Volbracht and A. Bredal Christensen (H. Lundbeck A/S, Valby, Denmark).

### 2.4 Viability and Cell Death Assays

Cell death was measured after 15 min propidium iodide (0.2 μg/mL) and Hoechst-33342 staining (2.5 μg/mL) at 37 °C employing Celígo® Imaging Cytometer (Nexcelom Bioscience) according to the manufacturer's manual. Apoptotic nuclear condensation was evaluated in Hoechst-33342 stained cells using Olympus IX81 microscope with a 20 × Olympus objective, Scan^R automated acquisition software (version 2.3.0.5) and analysis with ImageJ (version 1.48v). To evaluate clonogenic survival, cells were seeded at approximately 400 cells/well in 24-well plates, treated as indicated, stained with crystal violet-methanol for 15 min, washed three times in H_2_O, dried and analyzed with the colony verification application of the Celígo® Imaging Cytometer. Lysosomal membrane permeabilization was detected by staining paraformaldehyde-fixed cells on glass coverslides with antibodies recognizing LGALS-1 (Abcam, ab25138) and LAMP2 (Developmental Studies Hybridoma Bank, H4B4-S) followed by AlexaFluor488- or AlexaFluor594–coupled secondary antibodies (Molecular Probes, A21206 and A21203, respectively) as described previously (Aits et al., 2015). Hoechst-33342 staining was used to visualize the DNA. The samples were mounted with Pro-Long Gold anti-fade (Molecular Probes, P36935) and confocal fluorescent images were obtained with Carl Zeiss Axiovert LSM700 microscope with a 40 × Carl Zeiss objective and the Zen 2010 software.

### 2.5 Western Blot Analysis

Proteins separated in a 6–15% gradient SDS-PAGE and transferred to a nitrocellulose membrane were visualized with primary antibodies against MDR1 (Santa Cruz Biotechnology, sc-13131), alpha-tubulin (Abcam, ab15246) and GAPDH (AbD Serotec, MCA4740), horseradish peroxidase-conjugated secondary antibodies (anti-mouse, Dako, P0260; anti-rabbit, Vector Laboratories, PI-1000), and ECL Western blotting reagents (BIORAD, 170-5061) employing Luminescent Image Reader (Fujifilm, LAS-4000).

### 2.6 Rhodamine123 Assay

Cells were pre-treated for 1 h with 0.25 μg/mL Rhodamine123 (Invitrogen, 890808), treated as indicated for 2 h, washed in clear medium, stained with Hoechst-33342 and analyzed with the target 1 + 2 (Merge) application of the Celígo® Imaging Cytometer. Hoechst-33342 staining was used to verify equal cell number in the wells.

### 2.7 Statistical Analysis

The proportional hazards assumption for the registry-based study was assessed by testing for trends in the scaled Schoenfeld residuals. All analyses were performed in R version 3.0.2 using the packages survival (Therneau, 2014) and Epi (Carstensen et al., 2014). Level of significance was set to 5% in all analyses. The statistical significance of the experimental results was analyzed by a two-way ANOVA test followed by Dunnett's, Sidak's or Tukey's multiple comparisons tests (α = 0.05) using GraphPad Prism version 6.0e.

## 3 Results

### 3.1 A Screen for CADs That Kill NSCLC Cells

**Fig. 1:**
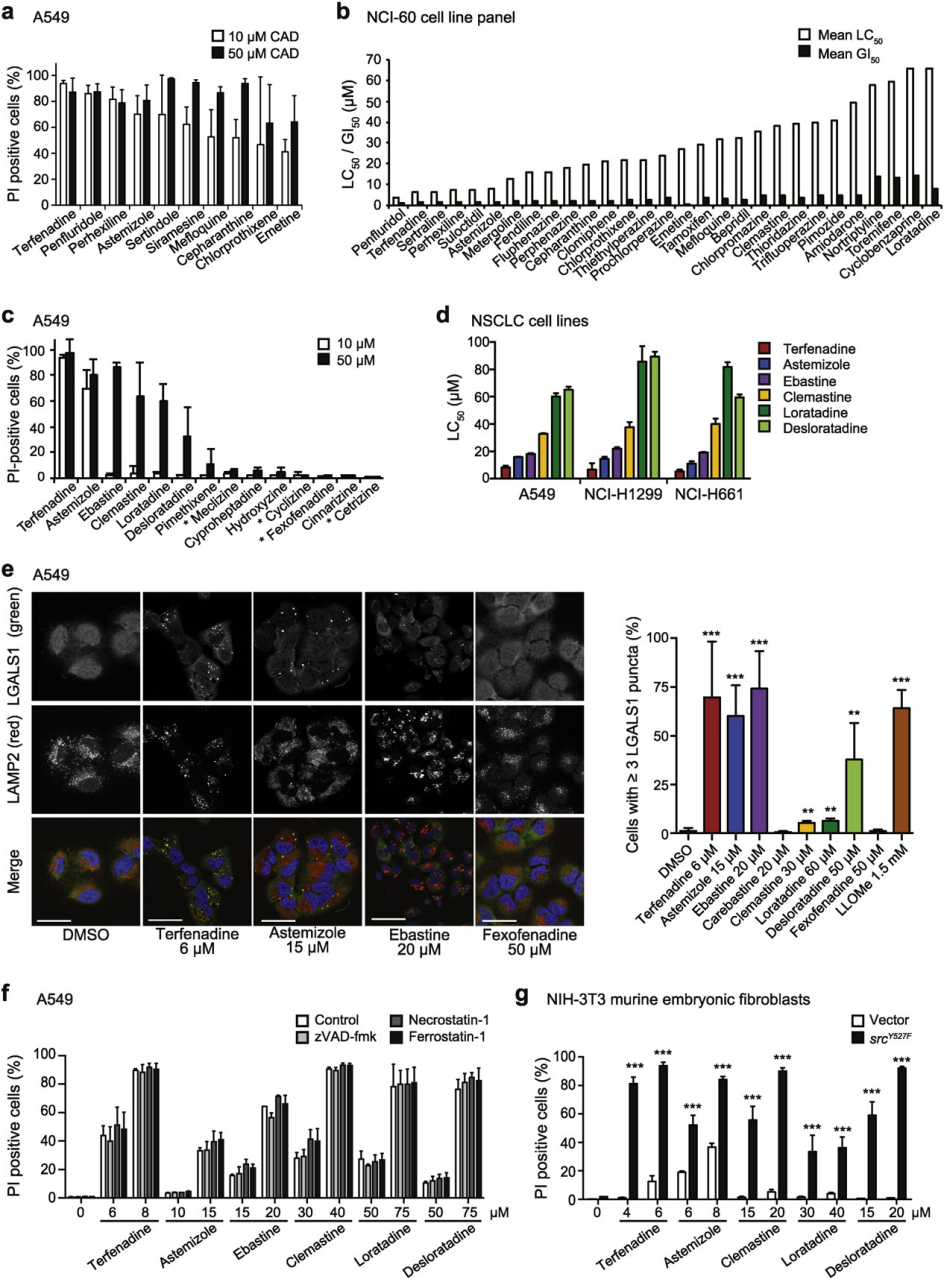
Identification and characterization of cytotoxic CADs. (a) Induction of cell death by the ten most cytotoxic drugs identified in a CAD library screen for PI exclusion after a 48 h treatment of A549 cells at 10 or 50 μM. (b) Mean LC_50_ and GI_50_ values for 29 of the hits from the CAD library screen in 60 human cancer cell lines (NCI-60 panel) treated for 48 h were extracted from the NCI homepage (NCI, 2015). (c) Death of A549 cells (PI exclusion) induced by treatment with 10 or 50 μM of indicated antihistamines for 48 h. Non-CAD antihistamines are marked with asterisks. (d) LC_50_ values for the selected CAD antihistamines in indicated NSCLC cell lines as analyzed by a 48 h PI exclusion assay. (e) Representative confocal images of A549 cells treated for 24 h as indicated and stained for LGALS1, lysosomal-associated membrane protein-2 (LAMP2) and DNA (Hoechst-33342) (*left*), and quantification of cells with ≥ 3 LGALS1 puncta (*right*). *L*-Leucyl-*L*-leucine *O*-Methyl ester (LLOMe) and fexofenadine served as positive and negative controls, respectively. A minimum of 100 randomly chosen cells *per* condition were counted. Scale bars, 50 μm. See also Supplemental Fig. 1c. (f) Death of A549 cells (PI exclusion) induced by a 48 h treatment with indicated concentrations of CAD antihistamines with or without a 1 h pre-treatment with 20 μM z-VAD-fmk, 10 μM necrostatin-1 or 1 μM ferrostatin-1. (g) Death of NIH-3T3-vector and c-src^Y527F^ cells (PI exclusion) induced by a 28 h treatment with indicated concentrations of CAD antihistamines. Error bars, SD for at least three independent triplicate experiments. *p < 0.05, **p < 0.01, ***p < 0.001 when comparing treated cells with untreated cells (e) or NIH-3T3-vector cells with NIH-3T3-c-src^Y527F^ (g) in a two-way ANOVA followed by Dunnett's (e) or Sidak's (g) multiple comparisons tests.

To identify clinically relevant drugs that could complement the existing NSCLC therapy, we screened a CAD library, containing 72 drugs selected based on their clinical safety profiles and reported ability to inhibit acid sphingomyelinase (Kornhuber et al., 2008, Kornhuber et al., 2010) for cytotoxicity against A549 NSCLC cells. The ten most potent drugs induced over 40% cell death at 10 μM and included two antihistamines, three antipsychotics, an antiangial, an antidepressant, an antimalarial, an antiprotozolal and an anti-inflammatory natural product (Fig. 1a). Fifty of the tested drugs induced over 40% cell death at 50 μM (Supplemental Table S5). The National Cancer Institute (NCI) homepage contains growth inhibition (GI_50_) and cytotoxicity (LC_50_) data for 29 of these compounds screened in a panel of 60 human tumor cell lines, including nine NSCLC cell lines (NCI, 2015). The mean GI_50_ and LC_50_ values for these CADs ranged from 0.02–14.2 μM and from 3.89–65.9 μM, respectively (Fig. 1b). All 29 CADs had strikingly similar dose response curves in the 60 cell lines tested indicating that their efficacy is not limited to NSCLC or cancers with specific genetic alterations (NCI, 2015). Based on the presence of two antihistamines among the top five hits and the favorable safety profiles of antihistamines, we focused our further investigations on this drug class.

### 3.2 CAD Antihistamines Destabilize Lysosomal Membranes

To further evaluate the anti-cancer activity of antihistamines, we first tested the cytotoxic potential of seven clinically relevant CAD antihistamines and four non-CAD antihistamines in A549 cells. In addition to terfenadine and astemizole that were among the top hits of our screen, four CAD antihistamines showed significant cytotoxicity against A549 cells at 50 μM, whereas the remaining three CAD antihistamines and all four non-CAD antihistamines failed to do so (Fig. 1c). Dose response studies of the six most effective CAD antihistamines revealed similar responses in three NSCLC cell lines (A549, NCI-H1299 and NCI-H661), where terfenadine had the lowest LC_50_ values between 5.4–8.2 μM, followed by astemizole (11.1–15.8 μM), ebastine (18.0–21.8 μM), clemastine (32.8–40.0 μM), desloratadine (59.5–89.4 μM) and loratadine (60.1–85.6 μM) (Fig. 1d and Supplemental Fig. S1a). These values are similar to the available mean LC_50_ values extracted from the NCI screen of 60 human cancer cell lines, *i.e.* 6.4 μM for terfenadine, 8.0 μM for astemizole, 38.3 μM for clemastine and 65.9 μM for loratadine (Fig. 1b). The active metabolites of terfenadine (fexofenadine) and ebastine (carebastine) showed no cytotoxicity against NSCLC cells, the primary metabolite of astemizole (*O*-desmethyl-astemizole) retained approximately half of the potency of the parent compound, and loratadine and its primary metabolite (desloratadine) showed similar potency (Fig. 1d; Supplemental Figs. S1a and b; data not shown).

Consistent with the reported ability of several CADs to induce lysosomal membrane permeabilization in other cancer cells (Petersen et al., 2013, Ostenfeld et al., 2008, Ellegaard et al., 2013, Sukhai et al., 2013), the cytotoxic CAD antihistamines induced lysosomal LGALS1 (galectin-1) puncta formation, a hallmark of lysosomal leakage (Aits et al., 2015), in A549, NCI-H1299 and NCI-H661 cells at concentrations around their LC_50_ values (Fig. 1e; Supplemental Fig. S1c; data not shown). Inhibition of apoptosis, necroptosis or ferroptosis by z-VAD-fmk, necrostatin-1 or ferrostatin-1, respectively, had no effect on the cell death induced by CAD antihistamines (Fig. 1f). Finally, the CAD antihistamines induced cell death in c-Src^Y527F^–transformed NIH-3T3 murine embryonic fibroblasts to a significantly higher extent than in the corresponding vector control cells (Fig. 1g). Taken together, these data show that CAD antihistamines induce cancer-specific lysosomal cell death in NSCLC cells.

### 3.3 Use of Astemizole and Loratadine is Associated With Reduced Cancer Mortality

**Fig. 2:**
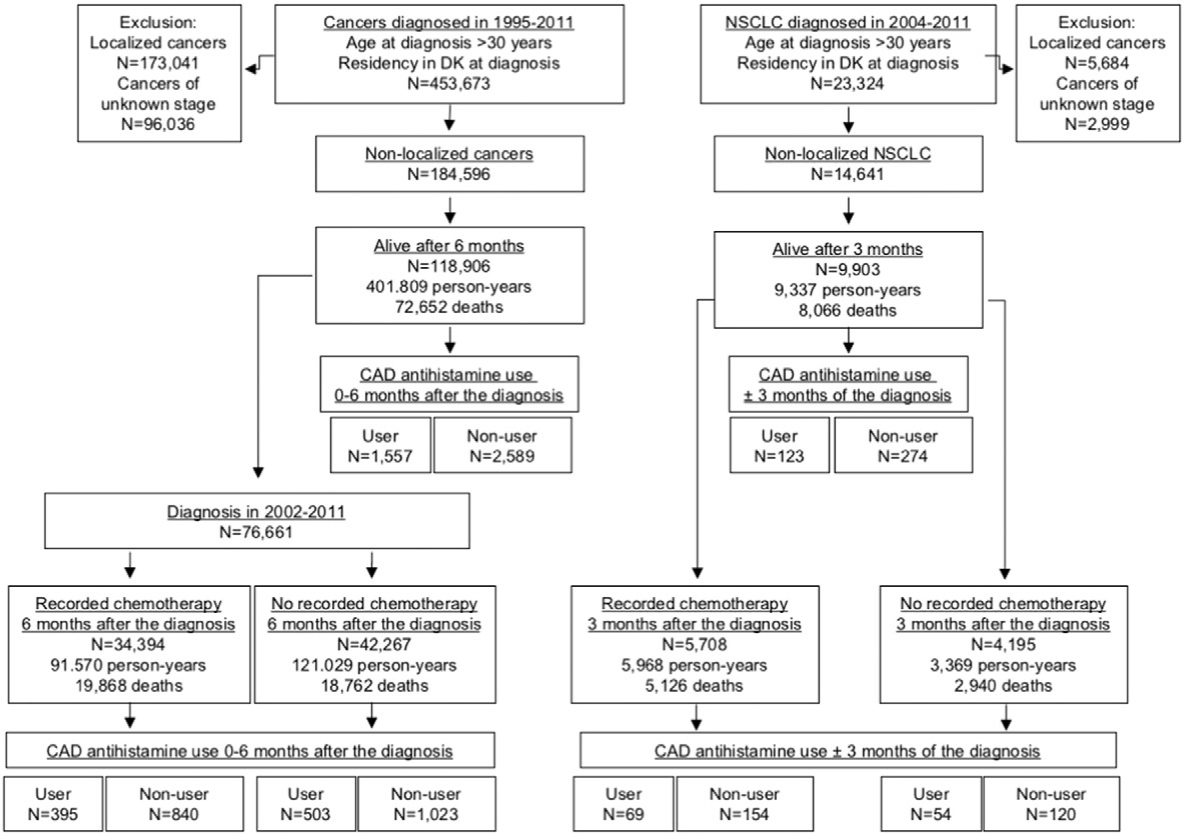
Consort flow diagram of the cohort studies.

Prompted by the cancer-specific cytotoxicity of CAD antihistamines, we conducted a nationwide pharmacoepidemiological cohort study of the association between the use of cytotoxic CAD antihistamines described above within six months after the diagnosis and mortality among all patients with any non-localized cancer (Fig. 2 and Supplemental Table S2). Astemizole or loratadine use was associated with significantly reduced all-cause mortality as compared with use of the non-CAD antihistamines, fexofenadine or cetirizine (Table 1; Supplemental Tables S3 and S6). The use of ebastine showed a similar tendency, use of terfenadine and desloratadine was without a significant effect, and clemastine use was associated with a significantly increased HR for mortality among patients with any non-localized cancer (Table 1). Suggestive of a prescribing bias, the use of clemastine increased over six-fold upon cancer diagnosis in our study cohort (see Supplemental Table S6 and Discussion).

When stratifying all patients with non-localized cancer according to the records of chemotherapy within six months after the diagnosis (available for patients diagnosed at 2002 or later), HRs for all-cause mortality among users of all eligible CAD antihistamines, except clemastine, were lower for patients with recorded chemotherapy than for those without such records (Table 1).

Low number of non-localized NSCLC patients hampered a similar analysis of NSCLC mortality for most CAD antihistamines. The aggressive nature of this disease further reduced the statistical power due to the high mortality during the first six months after the diagnosis. Thus, we redefined the drug exposure periods from six months after the diagnosis to three months before until three months after the diagnosis (Fig. 2). The use of the most commonly prescribed CAD antihistamine, loratadine, within this time period showed a statistically significant inverse association with mortality, and HRs for all-cause mortality among loratadine users were lower for patients with recorded concurrent chemotherapy than for those without such records (Table 2; Supplemental Tables S4 and S7). The effect appeared independent of the cancer histology because HRs for mortality were similar among patients with adenocarcinoma, squamous cell carcinoma and other types of NSCLC (Table 2).

### 3.4 Sub-micromolar Concentrations of CAD Antihistamines Revert MDR

**Fig. 3:**
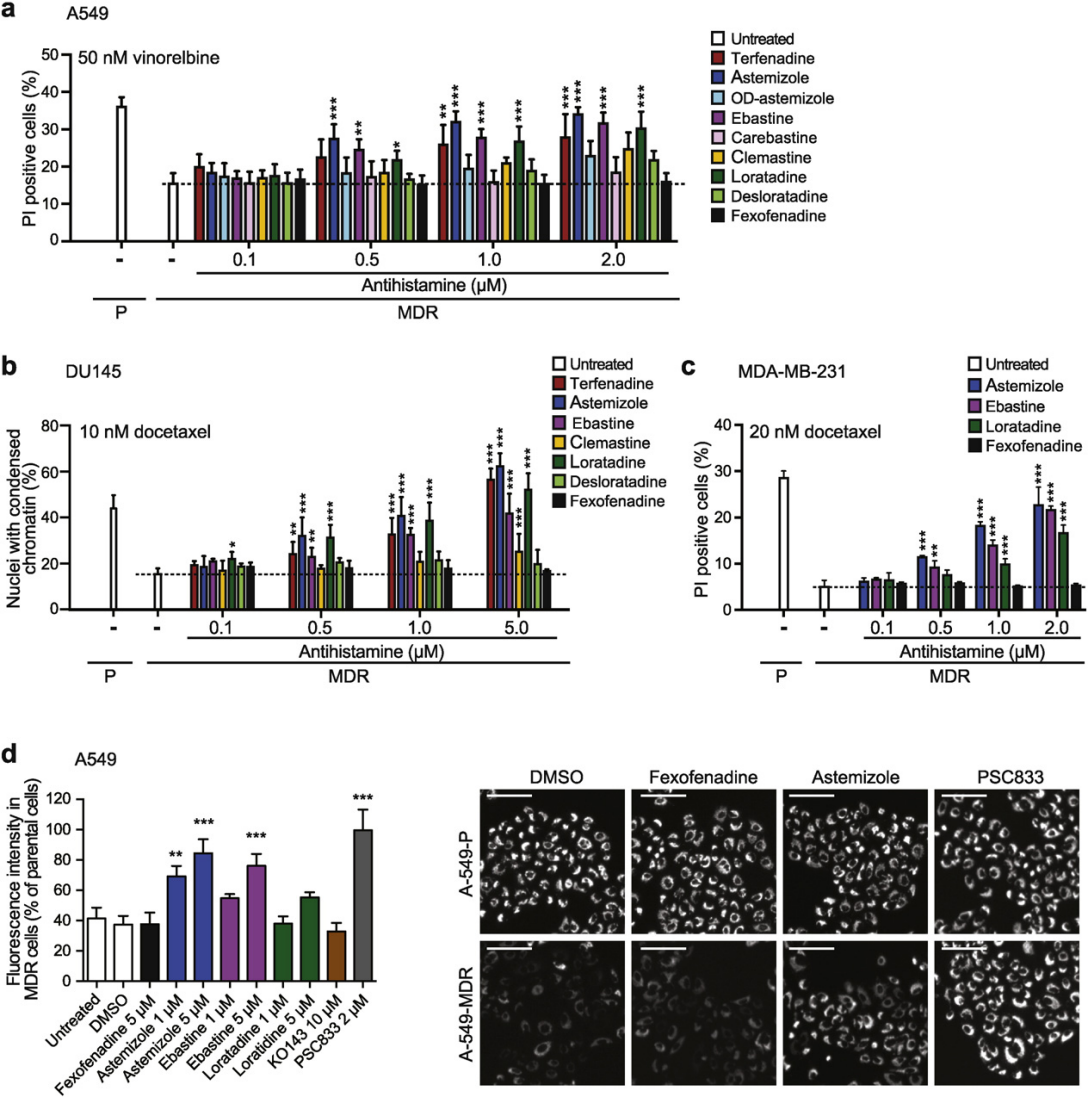
The ability of CAD antihistamines to re-sensitize MDR cancer cells to chemotherapy. (a–c) Death of parental (P) and multidrug resistant (MDR) A549 (a), DU145 (b) and MDA-MB-231 (c) cells (PI exclusion) induced by a 48 h treatment with indicated chemotherapeutics alone (white bars) or in combination with indicated concentrations of antihistamines. See also Supplemental Fig. S2c-e. (d) Fluorescence intensity in A549-P and A549-MDR cells treated with Rhodamine123 for 1 h prior to 2 h treatment with indicated concentrations of antihistamines or MDR inhibitors KO143 (ABCG2) or PSC833 (MDR1) was assessed with the Celígo® Imaging Cytometer (*left*). Representative images of selected conditions are shown (*right*). Scale bars, 100 μm. See also Supplemental Fig. S3. Error bars, SD for three independent, triplicate experiments. *p < 0.05, **p < 0.01, ***p < 0.001 when comparing MDR cells treated with and without antihistamines (a–c) or when comparing drug-treated and untreated cells (d) in a one-way (d) or two-way (a–c) ANOVA followed by Dunnett's multiple comparisons tests.

Clinically relevant doses of loratadine and astemizole result in plasma concentrations considerably lower than those required for effective inhibition of NSCLC cell growth or survival *in vitro* (Heykants et al., 1986, Hilbert et al., 1987). Thus, the putative anti-cancer effects observed above were probably not caused by CADs alone but rather by a combined effect of low concentrations of CADs and chemotherapy. MDR1-mediated resistance to chemotherapy represents one of the major barriers to positive long-term outcomes for this patient group (Chang, 2011), and several CADs have been reported to revert MDR1-associated drug resistance at micromolar concentrations (Jaffrezou et al., 1995, Petersen et al., 2013, Ellegaard et al., 2013). Thus, we tested whether low, clinically relevant concentrations of CAD antihistamines could re-sensitize MDR1-expressing NSCLC cells to chemotherapy. Because none of the three NSCLC cell lines used here had detectable MDR1 expression, we first created an MDR-variant of A549 cells by repeated treatments with increasing concentrations of vinorelbine (Supplemental Figs. S2a and b). Astemizole, ebastine and loratadine re-sensitized the obtained A549-MDR cells significantly to vinorelbine even at 500 nM, and terfenadine did so at 1 μM, whereas their primary metabolites OD-astemizole, carebastine, desloratadine and fexofenadine, respectively, failed to do so at concentrations up to 2 μM (Fig. 3a; Supplemental Fig. S2a). Similar MDR1-specific sensitization to docetaxel was observed in MDR1-expressing DU145-MDR prostate cancer cells and MDA-MB-231-MDR breast cancer cells treated with low concentrations of CAD antihistamines (Figs 3b and c; Supplemental Figs. S2d and e).

Astemizole and terfenadine have been reported to inhibit the efflux activity of MDR1 at IC_50_ of 1.3 and 1.4 μM, respectively (Schwab et al., 2003). Thus, we tested whether the other CAD antihistamines possessed similar ability at concentrations relevant for re-sensitization. Indicative of MDR1 activity, A549-MDR cells effectively effluxed the MDR1 substrate Rhodamine123 dye, which was completely inhibited by 2 μM PSC833 (MDR1 inhibitor) and by 50% by 1 μM astemizole (Fig. 3d; Supplemental Fig. S3). Ebastine and loratadine failed to inhibit the dye exclusion at 1 μM suggesting that CAD antihistamines can revert drug resistance also by mechanisms other than the direct inhibition of the efflux activity of MDR1 (Fig. 3d).

### 3.5 CAD Antihistamines and Chemotherapy Synergize to Induce Apoptotic and Lysosomal Cell Death

**Fig. 4:**
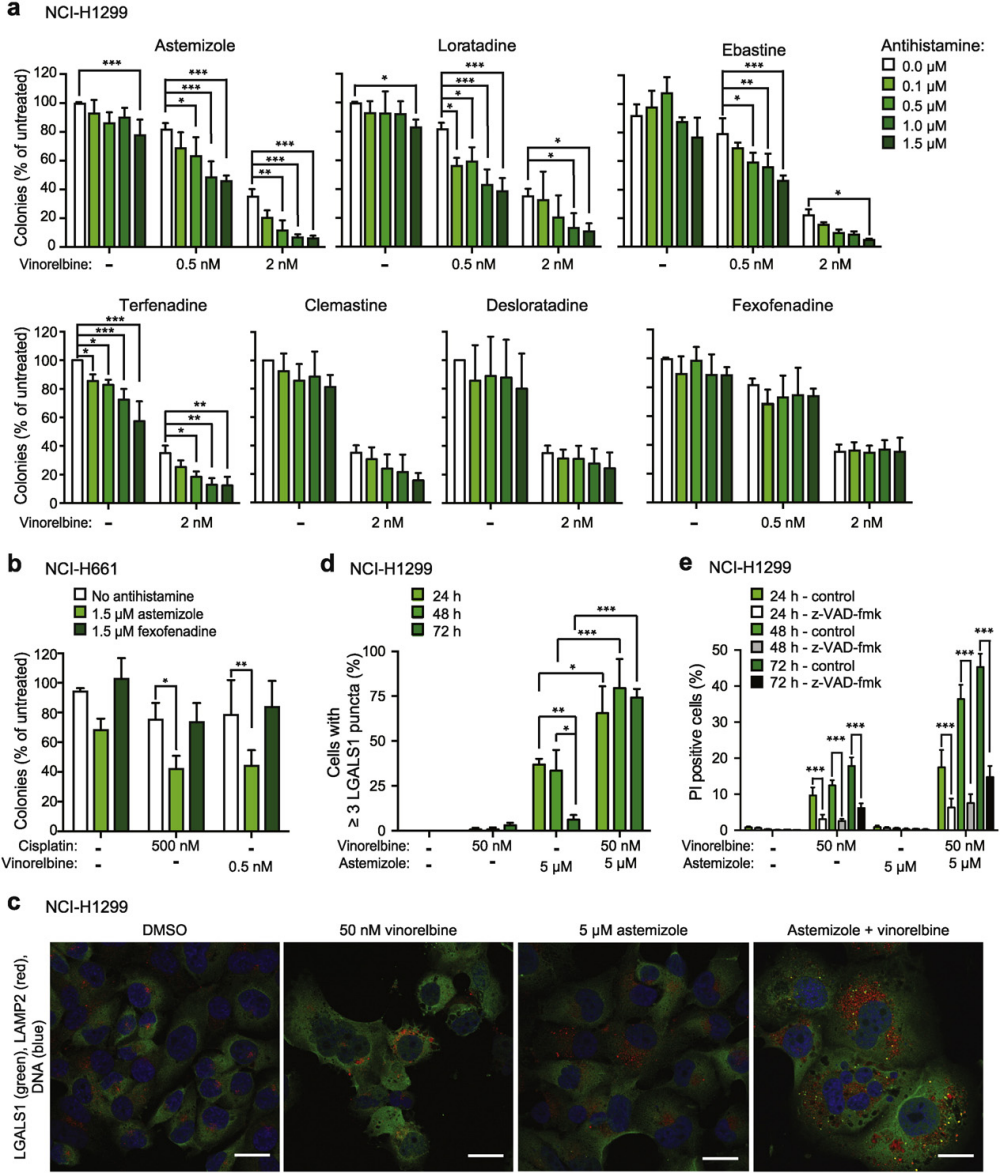
The ability of CAD antihistamines to sensitize NSCLC cells to chemotherapy. (a and b) Clonogenic survival of NCI-H1299 (a) and NCI-H661 (b) cells treated with indicated combinations of CADs and chemotherapy for four days. (c–e) Representative confocal images of NCI-H1299 cells treated for 72 h as indicated and stained for LGALS1, LAMP2 and DNA (Hoechst-33342) (c), and quantification of cells with ≥ 3 LGALS1 puncta (d) and cell death (e) after similar treatment for 24–72 h. When indicated, cells were pre-treated with 20 μM z-VAD-fmk for 1 h (e). Scale bars, 25 μm. A minimum of 50 cells *per* condition were counted in (d). Error bars, SD for a minimum or three (a, b and e) or 2–3 (d) independent, triplicate experiments. *p < 0.05, **p < 0.01, ***p < 0.001 when comparing cells as indicated in a two-way ANOVA followed by Dunnett's (a, b), Tukey's (d) or Sidak's (e) multiple comparisons tests.

To search for MDR1-independent mechanisms of synergy between CAD antihistamines and chemotherapy, we tested whether low non-toxic concentrations of CAD antihistamines enhanced the inhibitory effect of chemotherapy on clonogenic potential of NSCLC cells. Indeed, sub-micromolar concentrations of astemizole, ebastine, loratadine and terfenadine potentiated the inhibitory effect of vinorelbine on colony formation of NCI-H1299 cells, whereas clemastine, desloratadine and fexofenadine failed to do so (Fig. 4a). Similarly, astemizole, but not fexofenadine, sensitized NCI-H661 cells to subtoxic concentration of vinorelbine or cisplatin (Fig. 4b).

To investigate the mechanisms underlying the synergism between CADs and chemotherapy, we first analyzed lysosomal leakage by counting LGALS1 (galectin 1)-positive lysosomes in NCI-H1299 cells treated with suboptimal concentrations of astemizole and vinorelbine for 24–72 h. A non-toxic concentration of astemizole triggered a transient (24–48 h) accumulation of LGALS1 positive lysosomes, which were cleared at 72 h (Figs. 4c–d). Addition of vinorelbine at a concentration, which alone killed approximately 20% of the cells without disturbing lysosomal membrane integrity, enhanced astemizole-induced lysosomal damage (LGALS1 puncta formation) and inhibited the clearance of damaged lysosomes resulting in significantly enhanced cell death (Figs. 4c–e). Contrary to CAD-induced cell death that is independent of caspases (Fig. 1f), the pan-caspase inhibitor z-VAD-fmk inhibited approximately 60% of the cell death induced by vinorelbine alone or in combination with astemizole (Fig. 4e). These data suggest that the synergistic effect of astemizole and vinorelbine in NCI-H1299 cells results from the enhancement of both lysosomal and caspase-dependent cell death pathways.

## 4 Discussion

Data presented above suggest that addition of clinically relevant doses of well-tolerated CAD antihistamines to the standard cancer chemotherapy regiment improves cancer prognosis. This conclusion is based on statistically significant inverse associations between the use of loratadine, or the use of either loratadine or astemizole, and all-cause mortality among Danish patients with non-localized NSCLC or any non-localized cancer, respectively. Furthermore, ebastine use was associated with reduced all-cause mortality, albeit not statistically significant, in both study cohorts. Importantly, use of non-CAD antihistamines, fexofenadine and cetirizine, which have similar antihistamine effects and are prescribed for similar indications as CAD antihistamines, did not affect cancer mortality. Thus, the observed positive effect of CAD antihistamines is likely to be related to their CAD structure rather than their antihistamine effect or the disease they have been prescribed for.

Our pharmacoepidemiological study was inspired by the ability of CAD antihistamines to induce cancer-specific lysosomal cell death *in vitro*. The putative clinical benefit of CAD antihistamines is, however, not likely to be due to their direct cytotoxicity alone. Their LC_50_ and GI_50_ values are significantly higher than reported plasma concentrations achieved with recommended doses of these drugs, which range from undetectable for ebastine to 11 nM for astemizole and 68 nM for loratadine (Del Cuvillo et al., 2006). Instead, their ability to sensitize cancer cells to chemotherapy and revert MDR phenotype at sub-micromolar concentrations may explain the positive effects observed in our register-based study. This assumption is supported by the subgroup analyses showing that patients with records of chemotherapy within six months following the diagnosis of non-localized cancer or within three months of the diagnosis of non-localized NSCLC had additionally reduced HRs for all-cause mortality.

Interestingly, all three antihistamines emerging as putative anti-cancer drugs in our studies have extremely high apparent volumes of distribution (V_D_) ranging from 48 to over 100 L/kg for astemizole (Tillement, 2000), loratadine (Tillement, 2000) and ebastine (Del Cuvillo et al., 2006) (Table 3). High V_D_ values reflect the efficient distribution of drugs to tissues. Accordingly, the reported concentrations of astemizole in *e.g.* lungs, kidneys, liver and pancreas of Beagle dogs treated for six weeks with 1 mg/kg astemizole are over 1000-fold higher than the corresponding plasma concentrations (Tillement, 2000, Michiels et al., 1986). Notably, astemizole and other CADs, which per definition are weak bases, are likely to accumulate in acidic tumors even more efficiently than in healthy tissues with neutral pH. Data for tissue distribution of loratadine and ebastine are unfortunately not available, but their higher V_D_ values suggest even more efficient tissue distribution than observed for astemizole. On the other hand, the approximately 50-fold lower V_D_ (Tillement, 2000) and less efficient tissue distribution of terfenadine (Leeson et al., 1982) may explain the discrepancy between its potent anti-cancer activity *in vitro* and lack of effect in the pharmacoepidemilogical study. Contrary to the other CADs studied here, clemastine use was associated with increased cancer mortality. Notably, clemastine is commonly used in prevention and treatment of hypersensitivity reactions associated with cancer therapy at Danish hospitals and its use increased over six-fold after cancer diagnosis in our patient cohort. Such a prescription bias towards high-risk patients may thus explain the poor prognosis associated with the use of clemastine. It should also be noted that clemastine has a relatively low V_D_ value (Schran et al., 1996) (Table 3), and its ability to augment chemotherapy in parental and MDR cancer cells *in vitro* is inferior to that of astemizole, loratadine and ebastine. Over-the-counter sale of loratadine (< 35% of the total sale) and ebastine (< 25%) could create another potential source of bias. Such exposure misclassification is, however, considered negligible.

Taken together, the data presented here suggest that repurposing of safe and inexpensive CAD antihistamines to cancer therapy may enhance the anti-neoplastic response of chemotherapy especially in the case of microtubule-disturbing drugs. Further studies addressing the dose-responses and tissue distribution of CAD antihistamines and efficacy of various treatment combinations in pre-clinical animal models will hopefully pave the way for subsequent clinical trials in patients with distant stage NSCLC as well as other advanced cancers in near future.

## Funding Sources

This work was supported by grants from the European Research Council (Advanced grant number 340751), the Danish National Research Foundation (grant number DNRF125), the Danish Cancer Society (grant number R90-A5783), the Danish Medical Research Council (grant number DFF4004-00465), the Novo Nordisk Foundation (grant number NNF12OC0001341) and the Danish Cancer Research Foundation (project grant from 2011) to MJ.

## Conflict of Interest Statement

The authors disclose no potential conflicts of interest.

## Author Contributions

A.M.E. designed and performed most of the cell culture experiments, analyzed the data and contributed to the writing of the manuscript. C.D. designed and performed the analyses of all registry-based data. A.C.V. performed experiments presented in Fig. 3c and Fig. S2e. A.A. designed and performed the experiment presented in Fig. S1b. L.C. assisted in the design of the registry-based studies, N.H.T.P. and J.N. contributed to the design of cell culture experiments, J.S. designed the experiments and analyzed the data presented in Fig. 3c and Fig. S2e. A.M. and K.Ø. provided important insight into the clinical practice and possible confounding factors. S.F. designed the epidemiological analyses, analyzed the data and contributed to the writing the manuscript. M.J. designed the study, analyzed the biological data and wrote the first draft of the manuscript. All authors contributed to the final text and approved it.

## Figures and Tables

**Table 1:** Adjusted HRs and 95% CIs for mortality of patients with any non-localized cancer and ≥1 prescriptions of indicated CAD antihistamines within six months after the diagnosis compared with those with ≥1 prescriptions of non-CAD antihistamines (cetirizine or fexofenadine). See also Supplemental Tables S3 and S6.

Drug	HR^a^	2.5%	97.5%	P	N
Astemizole					
All patients^b^	0.67	0.46	0.98	0.040	38
Clemastine					
All patients^b^	1.32	1.08	1.60	0.006	154
With chemotherapy^c^	1.51	1.07	2.14	0.020	45
Without chemotherapy^c^	1.37	0.87	2.17	0.177	46
Desloratadine					
All patients^b^	0.94	0.79	1.13	0.524	280
with chemotherapy^c^	0.79	0.61	1.02	0.071	123
without chemotherapy^c^	0.97	0.73	1.28	0.832	150
Ebastine					
All patients^b^	0.82	0.62	1.09	0.181	87
with chemotherapy^c^	0.81	0.43	1.51	0.505	18
without chemotherapy^c^	0.94	0.58	1.53	0.806	38
Loratadine					
All patients^b^	0.90	0.82	0.99	0.042	854
with chemotherapy^c^	0.76	0.63	0.93	0.009	209
without chemotherapy^c^	0.85	0.70	1.04	0.125	270
Terfenadine					
All patients^b^	1.00	0.83	1.20	0.988	166

a The values are adjusted for the year of diagnosis, age, Charlson Comorbidity Index score, disposable income and use of aspirin, statins, NA-NSAIDs and ACEi-ARB (Supplemental Table S4).

b All patients diagnosed with any non-localized cancer in 1995–2011.

c Data stratified by available (2002 − 2012) records of chemotherapy (registered, N = 34,394 or non-registered, N = 42,267) within six months after the cancer diagnosis. This analysis was not applicable for astemizole and terfenadine, which were withdrawn from the market in 1999 and 2004, respectively.

**Table 2:** Adjusted HRs and 95% CIs for the mortality of patients with non-localized NSCLC and ≥ 1 prescriptions of indicated CAD antihistamines from three months before until three months after the diagnosis compared with those with ≥ 1 prescriptions of non-CAD antihistamine (cetirizine or fexofenadine). See also Supplemental Tables S4 and S7.

Drug	HR^a^	2.5%	97.5%	P	N
Clemastine	1.04	0.48	2.25	0.923	9
Desloratadine	1.10	0.79	1.54	0.554	47
Ebastine	0.63	0.26	1.54	0.310	7
Loratadine	0.69	0.49	0.96	0.030	60
With chemotherapy^b^	0.64	0.42	0.97	0.035	34
Without chemotherapy^b^	0.81	0.46	1.41	0.457	26
Adenocarcinoma^c^	0.62	0.35	1.08	0.094	22
Squamous cell carcinoma^c^	0.65	0.34	1.25	0.196	18
Other histology^c^	0.70	0.40	1.22	0.207	20

a The values are adjusted for the year of diagnosis, age, Charlson Comorbidity Index score, disposable income and use of aspirin, statins, NA-NSAIDs and ACEi-ARB (Supplemental Table S5).

b Data stratified according to available records of chemotherapy within three months after the cancer diagnosis.

c Data stratified according to NSCLC subtypes.

**Table 3:** Apparent volumes of distribution (V_D_) of selected antihistamines.

Drug	V_D_ (L/kg)	Reference
Astemizole	48	Tillement (2000)
Clemastine	11.4^a^	Schran et al. (1996)
Desloratadine	49	Molimard et al. (2004)
> 100	Del Cuvillo et al. (2006)
Ebastine	> 100	Del Cuvillo et al. (2006)
Loratadine	120	Tillement (2000)
Terfenadine	2.2–2.9	Tillement (2000)
Cetirizine	0.4	Tillement (2000)
0.5	Del Cuvillo et al. (2006)
Fexofenadine	5.6 ± 0.7	Tillement (2000)

a The V_D_ value given in liters (L) was converted to L/kg by dividing with an estimated average weight of 70 kg for the men included in the study.

## Appendix A Supplementary data

Supplemental Tables S1-S7 and Supplemental Figures S1-S3. Image 1
