# Supplementary material for: Repurposing Cationic Amphiphilic Antihistamines for Cancer Treatment
Source: eBioMedicine. 2016 Jun 7;9:130–9. doi: 10.1016/j.ebiom.2016.06.013 (PMC4972561; doi:10.1016/j.ebiom.2016.06.013)
Supplement: Supplementary file 1 — Supplemental Tables S1-S7 and Supplemental Figures S1-S3. [file mmc1.pdf]

Repurposing cationic amphiphilic antihistamines for cancer treatment

Supplemental Material:

- Supplemental Tables S1-S7
- Supplemental Figures S1-S3

Supplemental Table S1. Distribution of cancers diagnosed at non-localized stage in Denmark in 1995-2011 in main cancer groups or chemotherapy records (available from 2002) and use of listed CAD and non-CAD antihistamines among those alive six months after the diagnosis.

**Cancer groups (1995-2012)**	**Non-user[1]**	**Non-CAD user[2]**	**CAD user[3]**
Digestive organs	30,330	697	392
Breast	28,102	684	461
Respiratory system and thoracic organs	20,729	504	248
Male genital organs, including skin	11,440	173	108
Female genital organs, including skin	7,218	148	120
Lymphatic and haematopoietic tissue	2,929	75	58
Buccal cavity and pharynx	4,044	92	51
Urinary tract	3,783	74	38
Skin (melanoma)	1,925	46	23
Endocrine glands	686	12	8
Mesothelium and connective tissue	536	6	7
Eye, brain and other parts of CNS	367	9	9
Bones, joints and articular cartilage	58	0	0
Ill-defined and unspecificed cancer	2,613	69	34
All non-localized cancers (1995-2011)	114,760	2,589	1,557
**Chemotherapy records for all non-localized cancer (2002-2011)**			
Chemotherapy recorded	33,159	840	395
Chemotherapy not recorded	40,741	1,023	503
All non-localized cancers (2002-2011)	73,900	1,863	898

[1] Non-user of astemizole, clemastine, desloratadine, ebastine, loratadine, terfenadine, cetrizine and fexofenadine
[2] Cetrizine and fexofenadine
[3] Astemizole, clemastine, desloratadine, ebastine, loratadine or terfenadine

Supplemental Table S2. Registry data used in the analyses.

**The Danish Cancer Registry**			
All cancer (except nonmelanoma skin cancer); exclusion criterion before the date of index (first-time) cancer diagnosis		C00-C96 (except C44), B21, D32-D33, D35.2-D35.4, D42, D43, D44.3-D44.5	
Lung cancer		C34	
**Danish Prescription Registry**			
**Drug**		**Anatomical Therapeutic Chemical Classification**	
Antihistamines		R06AA04, R06AE07, R06AX11[1], R06AX12[1], R06AX13, R06AX22, R06AX26, R06AX27	
Aspirin		B01AC06 (75 mg), N02BA01 (100, 150 mg) N02BA01, N02BA51 (500 mg)	
Nonaspirin nonsteroidal anti-inflammatory drugs		M01A except M01AX	
Statins		C10AA	
ACE inhibitors		C09A, C09B	
Angiotensin II receptor blockers		C09C, C09D	
**National Patient Registry: Modified Charlson Comorbidity Index**			
**Disorder**	**Weight**	**ICD-8[2]**	**ICD-10[3]**
Myocardial infarction	1	410	I21;I22;I23
Congestive heart failure	1	427.09; 427.10; 427.11; 427.19; 428.99; 782.49	I50; I11.0; I13.0; I13.2
Peripheral vascular disease	1	440; 441; 442; 443; 444; 445	I70; I71; I72; I73; I74; I77
Cerebrovascular disease	1	430-438	I60-I69; G45; G46
Dementia	1	290.09-290.19; 293.09	F00-F03; F05.1; G30
Chronic pulmonary disease	1	490-493; 515-518	J40-J47; J60-J67; J68.4; J70.1; J70.3; J84.1; J92.0; J96.1; J98.2; J98.3
Connective tissue disease	1	712; 716; 734; 446; 135.99	M05; M06; M08; M09;M30;M31; M32; M33; M34; M35; M36; D86
Ulcer disease	1	530.91; 530.98; 531-534	K22.1; K25-K28
Mild liver disease	1	571; 573.01; 573.04	B18; K70.0-K70.3; K70.9; K71; K73; K74; K76.0
Diabetes (type1 and 2)	1	249.00; 249.06; 249.07; 249.09; 250.00; 250.06; 250.07; 250.09	E10.0, E10.1; E10.9; E11.0; E11.1; E11.9
Hemiplegia	2	344	G81; G82
Moderate or severe renal disease	2	403; 404; 580-583; 584; 590.09; 593.19; 753.10-753.19; 792	I12; I13; N00-N05; N07; N11; N14; N17-N19; Q61
Diabetes with end organ damage	2	249.01-249.05; 249.08 250.01-250.05; 250.08	E10.2-E10.8 E11.2-E11.8
Moderate or severe liver disease	3	070.00; 070.02; 070.04; 070.06; 070.08; 573.00; 456.00-456.09	B15.0; B16.0; B16.2; B19.0; K70.4; K72; K76.6; I85

[1] R06AX11 (astemizole) and R06AX12 (terfenadine) were withdrawn from the market due to risk of QTc interval prolongation and related arrhythmias in 1999 and 2004, respectively.
[2] ICD-8, International Classification of Diseases, Eighth Revision
[3] ICD-10, International Classification of Diseases, Tenth Revision

Supplemental Table S3. Description of the subjects included in the analyses for all non-localized cancers. Related to Tables 1 and S6.

Variable	Level	Non-user[1]	Non-CAD[2]	CAD[3]
**Astemizole**	Non-user	114760	2589	1519
	User	0	0	38
**Clemastine**	Non-user	114760	2589	1403
	User	0	0	154
**Desloratadine**	Non-user	114760	2589	1277
	User	0	0	280
**Ebastine**	Non-user	114760	2589	1470
	User	0	0	87
**Loratadine**	Non-user	114760	2589	703
	User	0	0	854
**Terfenadine**	Non-user	114760	2589	1391
	User	0	0	166
**CAD antihistamine[3]**	Non-user	114760	2589	0
	User	0	0	1557
**Cetirizine**	Non-user	114760	448	1557
	User	0	2141	0
**Fexofenadine**	Non-user	114760	2100	1557
	User	0	489	0
**Non-CAD antihistamine[2]**	Non-user	114760	0	1557
	User	0	2589	0
**Age category**	31-59	36944	876	547
	59-68	30949	701	400
	68-77	28203	607	360
	77-109	18664	405	250
**Charlson index**	0	82360	1646	1019
	1	20614	565	334
	2+	11786	378	204
**Aspirin**	Non-user	97266	2131	1311
	User	17494	458	246
**Statins**	Non-user	102941	2284	1396
	User	11819	305	161
**NANSAID**	Non-user	90235	1916	1161
	User	24525	673	396
**ACEi or ARB**	Non-user	96814	2087	1302
	User	17946	502	255
**Disposable income[4]**	1	25939	607	340
	2	28957	673	413
	3	19817	454	274
	4	17868	408	244
	5	22179	447	286

[1]Non-user of astemizole, clemastine, desloratadine, ebastine, loratadine, terfenadine, cetrizine and fexofenadine
[2]Cetrizine and fexofenadine
[3]Astemizole, clemastine, desloratadine, ebastine, loratadine or terfenadine
[5]Disposable income categories according to quintiles

Supplemental Table S4. Description of the subjects included in the analyses for non-localized NSCLC. Related to Tables 2 and S7.

Variable	Level	Non-user[1]	Non-CAD[2]	CAD[3]
**Astemizole**	Non-user	9506	274	123
	User	0	0	0
**Clemastine**	Non-user	9506	274	114
	User	0	0	9
**Desloratadine**	Non-user	9506	274	76
	User	0	0	47
**Ebastine**	Non-user	9506	274	116
	User	0	0	7
**Loratadine**	Non-user	9506	274	63
	User	0	0	60
**Terfenadine**	Non-user	9506	274	123
	User	0	0	0
**CAD antihistamine[3]**	Non-user	9506	274	0
	User	0	0	123
**Cetirizine**	Non-user	9506	70	123
	User	0	204	0
**Fexofenadine**	Non-user	9506	197	123
	User	0	77	0
**Non-CAD antihistamine[2]**	Non-user	9506	0	123
	User	0	274	0
**Age category**	31-59	2198	71	20
	59-68	3053	89	50
	68-77	2842	70	33
	77-109	1413	44	20
**Charlson index**	0	5506	142	54
	1	2407	74	35
	2+	1593	58	34
**Aspirin**	Non-user	7090	198	77
	User	2416	76	46
**Statins**	Non-user	7440	211	90
	User	2066	63	33
**NANSAID**	Non-user	6538	181	90
	User	2968	93	33
**ACEi or ARB**	Non-user	7348	195	85
	User	2158	79	38
**Disposable income[4]**	1	2815	71	38
	2	2732	87	37
	3	1587	43	21
	4	1198	25	10
	5	1174	48	17

[1]Non-user of astemizole, clemastine, desloratadine, ebastine, loratadine, terfenadine, cetrizine and fexofenadine
[2]Cetrizine and fexofenadine
[3]Astemizole, clemastine, desloratadine, ebastine, loratadine or terfenadine
[4]Disposable income categories according to quintiles from lowest (1) to highest (5)

Supplemental Table S5. CAD library screen for cytotoxicity against A549 cells.

	Cell death % ± SD[1]					
**Compound name**	**10 µM**	**SD**	**50 µM**	**SD**	**Company / Cat. #**	**CAS #**
Alverine citrate	2.2	0.3	8.1	4.0	Sigma / A0424	5560-59-8
Amiodarone HCl	6.6	4.5	69.9	9.7	Sigma / A8423	19774-82-4
Amitriptyline HCl	2.5	0.6	25.3	25.1	Sigma / A8404	549-18-8
Amlodipine besylate	2.0	0.5	55.1	9.1	Sigma / A5605	111470-99-6
Aprindine HCl	3.2	0.8	39.9	41.2	Sigma / A7606	33237-74-0
Astemizole	70.0	14.2	80.8	11.8	Sigma / A2861	68844-77-9
AY9944	5.7	1.2	81.4	16.2	Calbiochem / 190080	366-93-8
Benztropine mesylate	1.9	0.1	8.3	2.3	MP Biochemicals / 153626	132-17-2
Bepridil HCl	4.6	0.1	83.4	4.7	Sigma / B5016	74764-40-2
Biperiden HCl	3.7	1.7	62.0	50.0	Sigma / B5311	1235-82-1
Camylofine diHCl	1.9	0.2	5.6	2.8	Sigma / C4665	54-30-8
Carvedilol	7.6	1.6	87.4	7.9	Sigma / C3993	72956-09-3
Cepharanthine	52.1	14.0	93.8	3.7	LKT Laboratories / C1718	481-49-2
Chlorpromazine HCl	27.7	30.6	91.1	4.6	Sigma / C8138	69-09-0
Chlorprothixene	46.6	52.2	63.2	29.7	Sigma / C1671	6469-93-8
Cinnarizine	6.1	7.8	1.8	0.3	Sigma / C5270	298-57-7
Clemastine fumarate	27.5	42.4	63.1	35.6	Sigma / SML0445	14976-57-9
Clofazimine	2.8	0.1	21.9	5.0	Sigma / C8895	2030-63-9
Clomiphene citrate	30.0	2.4	88.7	7.7	Sigma / C6272	50-41-9
Cloperastine HCl	2.5	0.3	53.5	38.4	Sigma / C2040	14984-68-0
Conessine	5.0	1.0	11.7	2.4	Sigma / SML0095	546-06-5
Cyclobenzaprine HCl	2.7	0.7	55.4	13.7	Sigma / C4542	6202-23-9
Cyproheptadine HCl sesquihydrate	2.2	0.8	5.8	2.8	Sigma / C6022	41354-29-4
Desipramine HCl	3.6	2.4	30.5	19.7	Sigma / D3900	58-28-6
Desloratadine	2.5	0.4	32.6	23.0	Sigma / D1069	100643-71-8
Dicyclomine HCl	2.3	1.3	33.8	53.7	Sigma / D7909	67-92-5
Dimebolin HCl	2.0	0.8	4.7	4.0	Cayman Chemicals / 10011349	97657-92-6
Drofenine HCl	2.1	0.1	7.7	3.9	Sigma / D7402	548-66-3
Emetine diHCl hydrate	41.0	9.6	64.2	20.2	Sigma / E2375	7083-71-8
Ethopropazine	2.3	0.6	18.3	18.4	Sigma / E5406	1094-08-2
Fendiline HCl	11.6	1.8	87.3	6.0	Sigma / F7265	13636-18-5
Flunarizine diHCl	3.5	0.4	48.6	10.8	Sigma / F8257	30484-77-6
Fluoxetine HCl	2.9	0.5	89.1	8.9	Sigma / F132	56296-78-7
Flupentixol diHCl	25.4	46.9	76.5	32.8	EPRS[2] / Y0000064	2413-38-9
Fluphenazine diHCl	5.5	1.4	94.8	3.6	Sigma / F4765	146-56-5
Hydroxyzine diHCl	1.9	0.3	5.2	3.3	Sigma / H8885	2192-20-3
Levomepromazine HCl	2.8	1.1	82.1	20.0	EPRS[2] / L0500000	1236-99-3

Lidoflazine	13.2	11.1	87.5	8.8	Sigma / L9668	3416-26-0
Lofepramine HCl	3.1	0.2	19.1	4.8	BPCRS[3] / 697	26786-32-3
Loperamide HCl	2.9	0.2	22.2	5.2	Sigma / L4762	34552-83-5
Loratadine	3.4	1.2	60.0	13.5	Sigma / L9664	79794-75-5
Maprotiline HCl	2.4	0.0	88.8	10.0	Sigma / M9651	10347-81-6
Mefloquine HCl	52.6	21.0	86.6	4.7	Sigma / M2319	51773-92-3
Metergoline	3.3	0.7	90.1	5.0	Sigma / M3668	17692-51-2
Metixene HCl	3.7	1.0	95.2	1.8	EPRS[2] / M180800	7081-40-5
Metoprolol tartrate	1.6	0.2	2.0	0.8	Sigma / M1830000-1EA	56392-17-7
Mibefradil diHCl hydrate	13.2	16.1	93.4	1.8	Sigma / M5441	116666-63-8
Norfluoxetine HCl	2.6	0.8	86.2	4.3	Sigma / F133	57226-68-3
Nortriptyline HCl	3.4	1.7	89.4	2.8	Sigma / N7261	894-71-3
Penfluridol	85.9	6.5	87.5	6.2	Sigma / P3371	26864-56-2
Perhexiline maleate	81.6	9.4	78.8	10.1	Sigma / SML0120	6724-53-4
Perphenazine	17.0	21.8	92.9	2.2	Sigma / P6402	58-39-9
Pimethixene maleate	2.0	0.3	11.0	12.2	Sigma / P4545	13187-06-9
Pimozide	25.3	35.1	79.2	11.5	Sigma / P1793	2062-78-4
Prenylamine	11.9	2.1	83.5	6.8	Toronto Research Chemicals / P712800	390-64-7
Prochlorperazine dimaleate	7.7	10.9	67.4	37.2	Sigma / P9178	84-02-6
(R)-(+)-Propranolol HCl	3.5	3.4	5.9	5.7	Sigma / P0689	13071-11-9
Protriptyline HCl	4.2	2.2	56.6	32.9	Sigma / P8813	1225-55-4
Sertindole	69.9	30.2	97.3	0.7	Sigma / S8072	106516-24-9
Sertraline HCl	5.8	2.7	94.8	2.4	Sigma / S6319	79559-97-0
Siramesine	62.4	13.3	94.5	2.2	H. Lundbeck A/S	147817-50-3
Solasodine	28.6	5.4	53.0	25.9	Aldrich Chemistry / R204536	126-17-0
Suloctidil	7.0	2.3	81.6	7.0	Sigma / S9384	54767-75-8
Tamoxifen citrate	3.4	0.7	94.5	1.9	Sigma / T9262	54965-24-1
Terfenadine	93.8	2.4	87.1	10.8	Sigma / T9652	50679-08-8
Thiethylperazine maleate	10.7	8.7	97.3	0.2	USP Reference Standard / 1658008	1179-69-7
Thioridazine HCl	25.5	45.1	89.0	7.4	Sigma / T9025	130-61-0
Tomatidine HCl	7.3	2.2	76.2	1.2	Sigma / T2909	6192-62-7
Toremifene citrate	3.3	0.5	92.9	3.3	Sigma / T7204	89778-27-8
Trifluoperazine diHCl	6.8	7.0	94.4	3.8	Sigma / T8516	440-17-5
Zolantidine	3.5	1.2	57.8	25.1	Tocris / 1070	104076-39-3
Zuclopenthixol HCl	26.6	44.5	72.3	33.4	BPCRS[3]	58045-23-1

[1]A549 cells were treated with indicated CAD concentrations for 48 h and cell death was measured by propidium iodide exclution assay. Values represent mean ± SD for a minimum of three independent, triplicate experiments.
[2]European Pharmacopoeia Reference Standard.
[3]British Pharmacopoeia Chemical Reference Substance.
[4]Siramesine was kindly provided by Dr. Christiane Volbracht, Lundbeck A/S.

Supplemental Table S6. Adjusted hazard ratios for all-cause mortality among patients with any non-localized cancer and ≥1 prescriptions of indicated antihistamines within six months after the diagnosis. Related to Table 1.

Drug	HR[1]	2.5%	97.5%	*P*	N	N (pre)[2]
**Astemizole**	0.68	0.47	0.98	0.041	38	52
**Clemastine**	1.33	1.10	1.60	0.004	154	21
**Desloratadine**	0.95	0.80	1.13	0.554	280	299
**Ebastine**	0.83	0.63	1.10	0.189	87	87
**Loratadine**	0.91	0.83	0.99	0.026	854	828
**Terfenadine**	1.00	0.84	1.20	0.954	166	131
**All CADs[3]**	0.94	0.88	1.01	0.070	1557	1418
**All CADs except clemastine[4]**	0.91	0.85	0.97	0.006	1414	1403
**Cetirizine[5]**	1.03	0.97	1.09	0.317	2141	1677
**Fexofenadine[5]**	0.90	0.80	1.02	0.110	469	494
**All non-CADs[5,6]**	1.01	0.96	1.06	0.798	2589	2171

[1]The values are adjusted for the year of diagnosis, age, Charlson score, disposable income and use of aspirin, statins, NANSAIDs and ACEi or ARB (Supplemental Table S3).
[2]Number of users within six months before the diagnosis.
[3]Astemizole, clemastine, desloratadine, ebastine, loratadine or terfenadine
[4]Clemastine was excluded from the "All CADs" group due to the observed prescription bias.
[5]Users of listed CAD antihistamines were excluded.
[6]Cetrizine or fexofenadine

Supplemental Table S7. Adjusted hazard ratios for all-cause mortality among patients with non-localized NSCLC and one or more prescriptions of indicated antihistamines within three months before and after the diagnosis. Related to Table 2.

Drug	HR[1]	2.5%	97.5%	*P*	N
**Clemastine**	1.07	0.50	2.29	0.864	9
**Desloratadine**	1.14	0.84	1.54	0.405	47
**Ebastine**	0.65	0.27	1.56	0.334	7
**Loratadine**	0.71	0.52	0.96	0.027	60
**All CADs[3]**	0.87	0.71	1.07	0.179	116
**All CADs except clemastine[4]**	0.86	0.70	1.06	0.154	114
**Cetirizine[5]**	0.95	0.81	1.12	0.598	204
**Fexofenadine[5]**	1.17	0.91	1.50	0.227	77
**All non-CADs[5,6]**	1.03	0.90	1.18	0.685	274

[1]The values are adjusted for the year of diagnosis, age, Charlson score, disposable income and use of aspirin, statins, NANSAIDs and ACEi or ARB (Supplemental Table S5).
[2]Number of users within six months before the diagnosis.
[3]Astemizole, clemastine, desloratadine, ebastine, loratadine or terfenadine
[4]Clemastine was excluded from the “All CADs” group due to the observed prescription bias.
[5]Users of listed CAD antihistamines were excluded.
[6]Cetrizine or fexofenadine

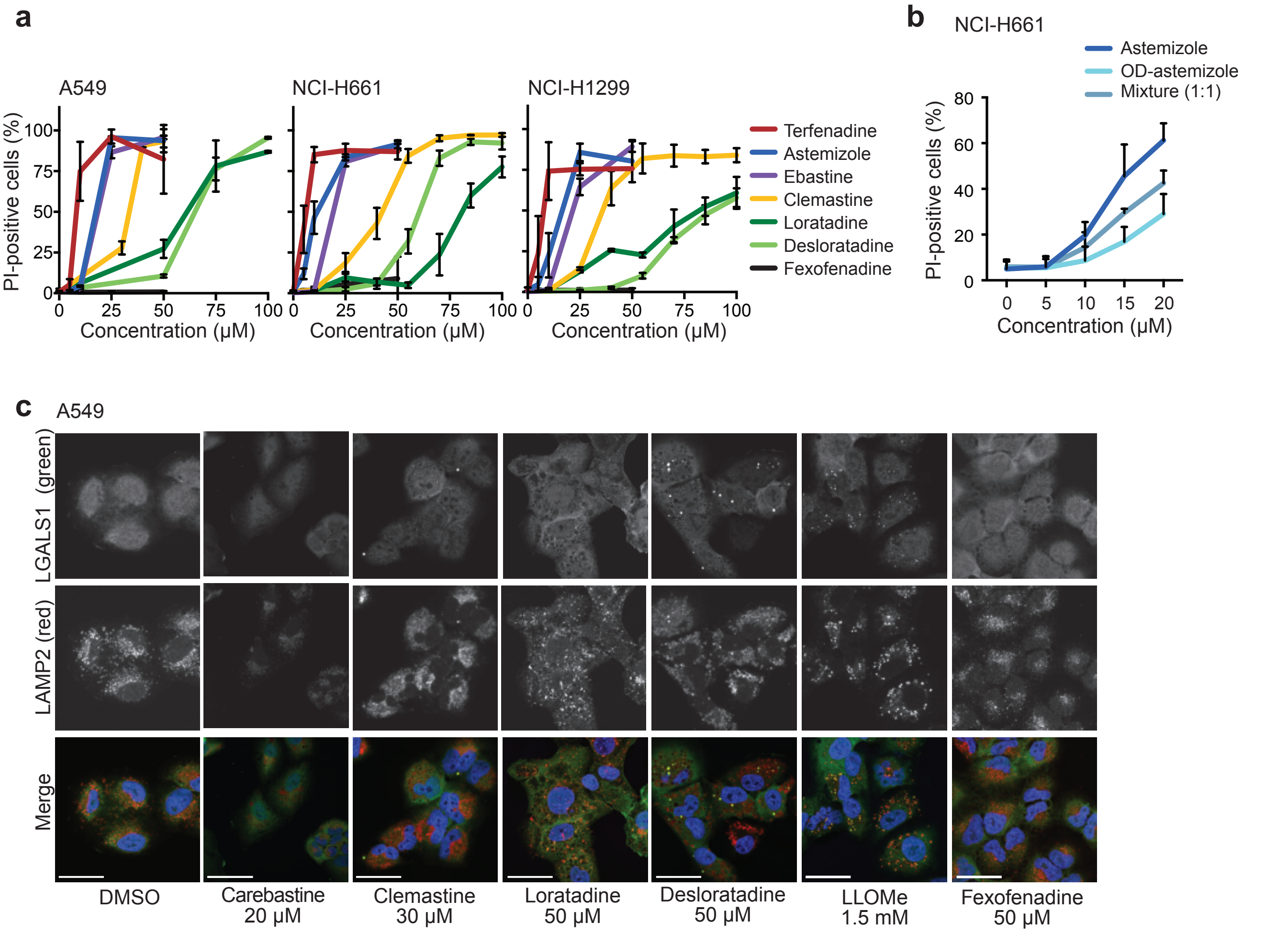

Supplemental Fig S1. Cytotoxicity and LMP induction by CADs (Related to Fig 1)
(a) Dose-response curves for the 48 h cytotoxicity (PI exclusion) of the indicated CAD antihistamines in A549, NCI-H661 and NCI-H1299 cells. A non-CAD antihistamine fexofenadine served as a negative control.
(b) Death of NCI-H661 cells (PI exclusion) induced by treatment with indicated doses of astemizole, O-desmethyl-astemizole (OD-astemizole) or their mixture (1:1) for 48 h.
(c) Representative confocal images of A549 cells treated for 24 h as indicated and stained for LGALS1, LAMP2 and DNA (Hoechst-33342). Lysosomal LGALS1 puncta are indicative of lysosomal damage. Quantification of the data is shown in Figure 1C. Scale bars, 50 µm
Error bars, SD for at least three independent triplicate experiments.

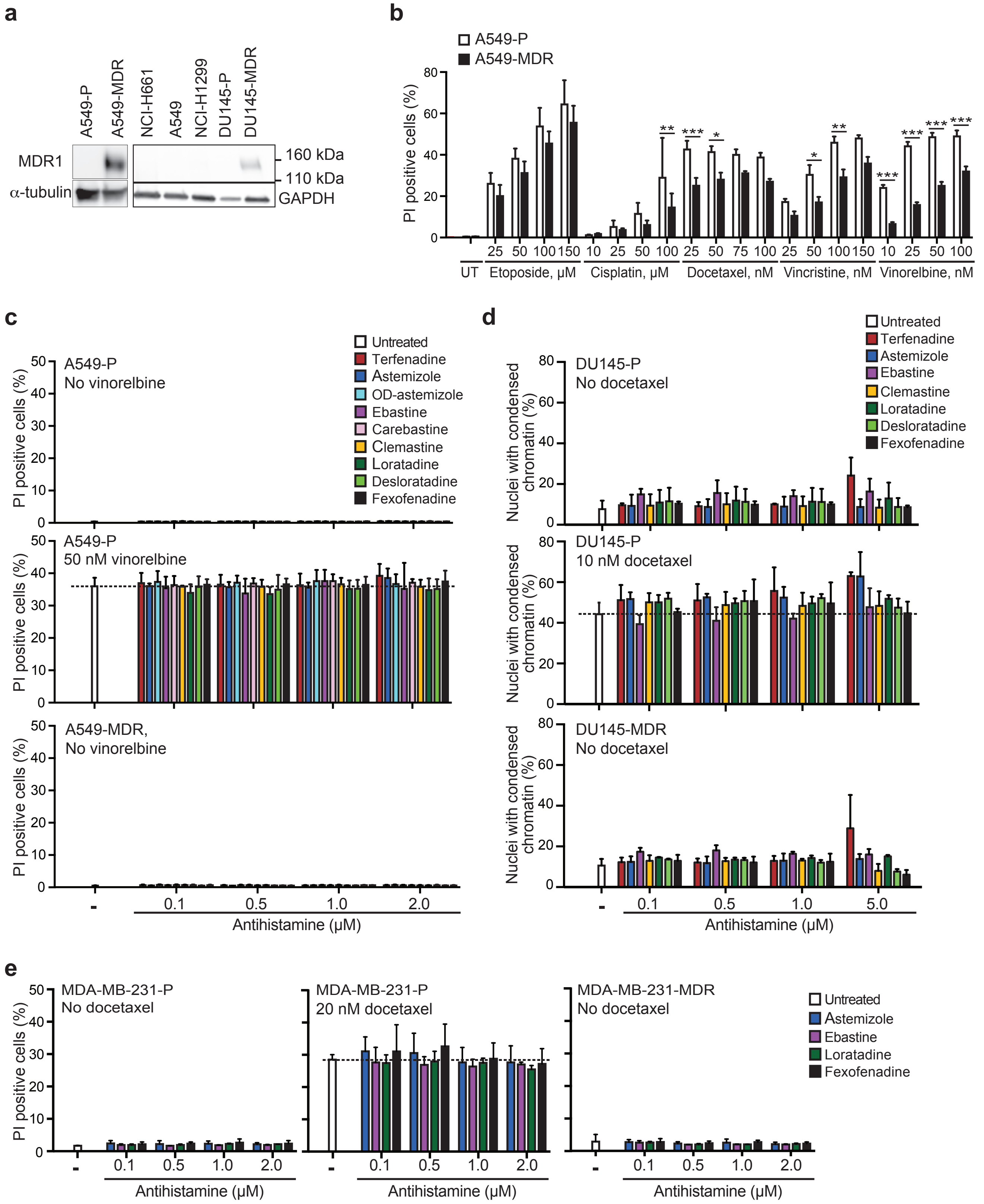

Supplemental Fig S2. CAD antihistamines sensitize MDR cells to chemotherapy (Related to Fig 2)
(a) Representative immunoblots (n=3) of MDR1 in lysates of indicated cells. α-tubulin and GAPDH served as loading controls.
(b) Death of parental (P) and multidrug resistant (MDR) A549 cells (PI exclusion) treated as indicated for 72 h.
(c - e) **.** Death of parental (P) and multidrug resistant (MDR) A549 (c), DU145 (d) and MDA-MB-231 (e) cells (PI exclusion) treated for 48 h with indicated chemotherapeutic drugs alone (white bars) or in combination with indicated concentrations of antihistamines.
Error bars, SD for three independent, triplicate experiments.
*p<0.05, **p<0.01, ***p<0.001 when comparing parental and MDR cells in a two-way ANOVA followed by Sidak's multiple comparisons test.

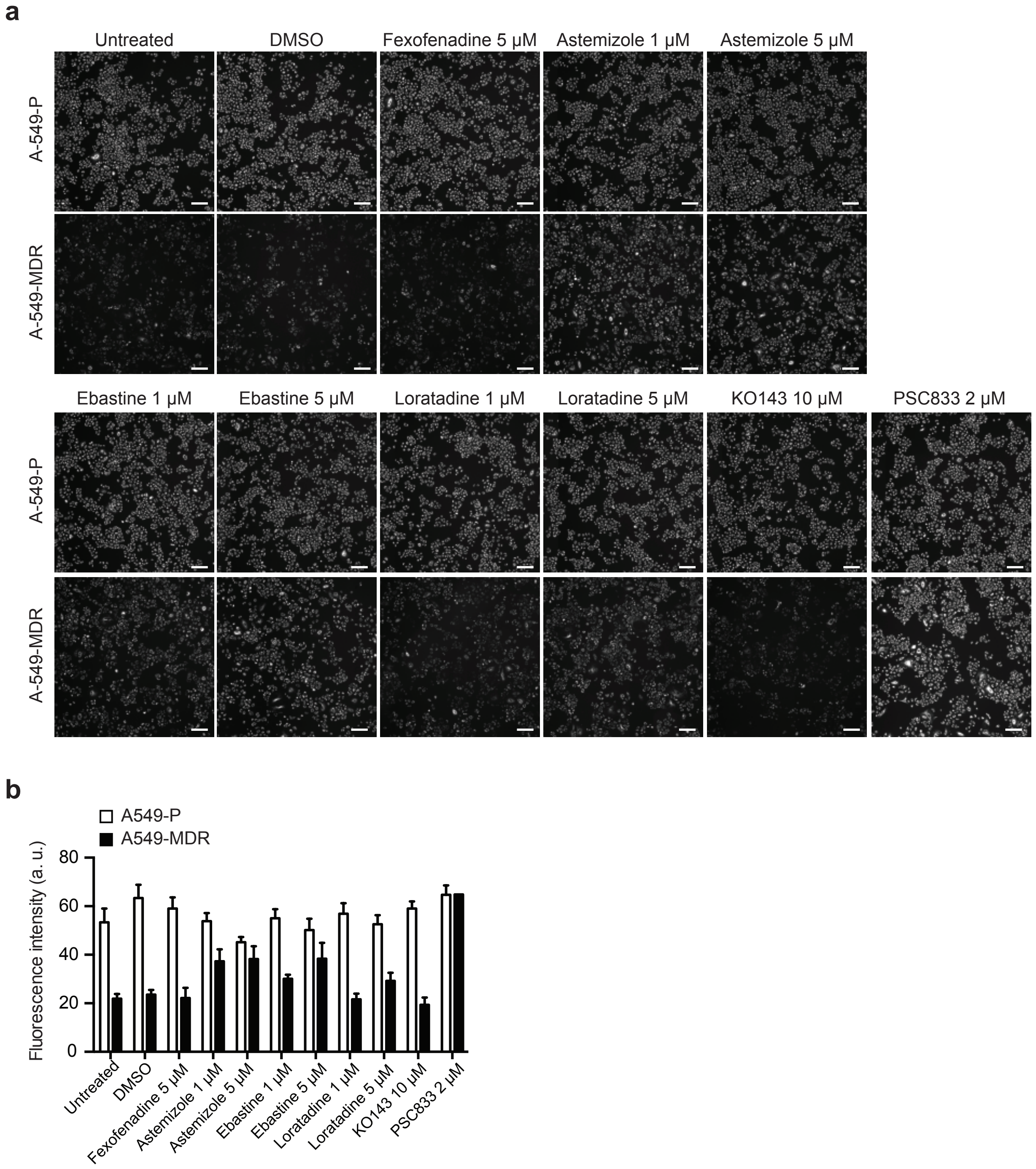

Supplemental Fig S3: Effect of antihistamines on MDR1-mediated efflux of Rhodamine123
(Related to Fig 3)
(a) Representative images of A549-P and A549-MDR cells treated with Rhodamine123 for 1 h prior to 2 h treatment with indicated doses of antihistamines or MDR inhibitors KO143 (ABCG2) or PSC833 (MDR1). Scale bars, 100 μm.
(b) The red fluorescence intensity of cells in (a) was assessed with the Celígo® Imaging Cytometer.
